# The great masquerader: primary vitreoretinal lymphoma presenting as white dot syndrome

**DOI:** 10.22336/rjo.2026.40

**Published:** 2026

**Authors:** Mihai Luca Cioboată, Ioana Tofolean, Suher Abduraman, Bogdana Maliș, Radu Burcea, Miruna Cioboată

**Affiliations:** 1 “Prof. Dr. Mircea Olteanu” Clinical Institute of Ophthalmological Emergencies, Bucharest, Romania; 2 “Carol Davila” University of Medicine and Pharmacy, Bucharest, Romania

**Keywords:** primary vitreoretinal lymphoma, primary central nervous system lymphoma, masquerade syndrome, white dot syndrome, COVID-19, PVRL = primary vitreoretinal lymphoma, CNS = central nervous system, WDS = white dot syndrome, MEWDS = multiple evanescent white dot syndrome, MTX = methotrexate, BCVA = best-corrected visual acuity, IOP = intraocular pressure, RAPD = relative afferent pupillary defect, LE = left eye, RE = right eye, PCNSL = primary central nervous system lymphoma, FAF = fundus autofluorescence, RPE = retinal pigment epithelium, OCT = optical coherence tomography

## Abstract

**Introduction:**

Primary vitreoretinal lymphoma (PVRL) is a rare and aggressive subtype of intraocular lymphoma, most commonly associated with primary central nervous system lymphoma (PCNSL). It frequently mimics inflammatory retinal diseases and is therefore considered an important masquerade syndrome in ophthalmology. Early diagnosis remains challenging because clinical manifestations can resemble entities within the white dot syndromes (WDS) spectrum. We report a case initially diagnosed as white dot syndrome, followed by the diagnosis of PCNSL, in which later ocular progression led to the diagnosis of PVRL.

**Case presentation:**

A 57-year-old male presented with painless, progressive visual loss in the left eye (LE) starting in July 2021. At that time, he was diagnosed at another center with multiple evanescent white dot syndrome (MEWDS) and treated with systemic corticosteroids, with subsequent improvement of visual symptoms after a single treatment course. His medical history was significant for COVID-19 infection in September 2021, followed one month later by neurological symptoms. Brain imaging revealed a left frontal tumor, and surgical resection established the diagnosis of PCNSL (non-Hodgkin B-cell lymphoma). The patient subsequently underwent systemic and intrathecal methotrexate (MTX) therapy combined with whole-brain radiotherapy. One year later, ophthalmologic reevaluation at our center revealed a best-corrected visual acuity (BCVA) of 20/20 in the right eye (RE) and motion in the left eye (LE). The LE showed a relative afferent pupillary defect (RAPD), vitreous cells, and multiple subretinal white-yellow lesions, including subfoveal involvement, associated with peripheral pigmentary changes. Given the patient’s medical history, the findings raised suspicion for intraocular lymphoma. A diagnostic vitrectomy was subsequently performed, confirming PVRL. Intravitreal MTX therapy was initiated. Despite treatment, BCVA in the LE was counting fingers, and extensive chorioretinal atrophy was observed. Although subtle changes in the central outer retinal layers were observed in the RE, BCVA was 20/25. The patient subsequently underwent autologous stem cell transplantation. However, the clinical course was further complicated by a relapse of PCNSL and a severe Escherichia coli sepsis.

**Discussion:**

Primary vitreoretinal lymphoma is well known for its ability to masquerade as chronic posterior uveitis or inflammatory chorioretinal diseases, including WDS. The presence of vitritis combined with multifocal subretinal infiltrates should raise suspicion, particularly in patients with a history of PCNSL. Corticosteroid responsiveness may further obscure the diagnosis by temporarily improving inflammatory-like manifestations. Because up to 80% of patients with PVRL develop CNS involvement or present with CNS disease first, ophthalmologic surveillance is essential. Multimodal imaging and a high index of suspicion are critical for timely diagnosis.

**Conclusion:**

Primary vitreoretinal lymphoma should be considered in the differential diagnosis of white dot-like retinal lesions, especially in patients with a history of CNS lymphoma. Recognition of this masquerade presentation is essential to avoid diagnostic delay and to ensure prompt multidisciplinary management.

## Introduction

Primary vitreoretinal lymphoma (PVRL) is a rare ocular malignancy, with an incidence of 0.5-2 cases per million. However, its reported frequency has increased in recent decades due to improved diagnostic methods, greater disease awareness, longer life expectancy, and a higher prevalence of immunosuppression [1,2]. It represents a heterogeneous and often underrecognized group of tumors, frequently misdiagnosed because of its subtle presentation and ability to mimic other ocular conditions, which also limits research and standardized management [3]. Central nervous system (CNS) involvement is common, occurring in 60-80% of cases, and a significant proportion of patients with PVRL will develop or present with CNS disease during the course of illness [4,5].

Primary vitreoretinal lymphoma commonly presents with floaters and progressive vision loss, often accompanied by characteristic retinal findings, including multifocal creamy-white lesions and a “leopard-skin” pattern [6]. It is an aggressive malignancy that frequently involves both eyes, with vitreous infiltration observed in most cases [7]. Primary vitreoretinal lymphoma should be suspected in elderly or immunosuppressed patients with visual symptoms that fail to respond to corticosteroid therapy [8]. Diagnosis is often challenging due to its variable presentation, as it can mimic a wide range of ocular conditions, including uveitis, viral retinitis, macular degeneration, white dot syndrome (WDS), and other inflammatory or infectious diseases. Therefore, careful clinical assessment and attention to subtle diagnostic clues are essential for early and accurate diagnosis [5].

Multiple evanescent white dot syndrome (MEWDS) is a rare, idiopathic, self-limiting inflammatory disorder of the outer retina that typically affects young women, often after a viral illness. It presents with foveal granularity and small gray-white lesions at the posterior pole, usually sparing the periphery [9]. Although MEWDS is typically benign, its clinical and imaging features may overlap with those of PVRL, creating diagnostic confusion. Both entities can present with gray-white outer retinal lesions and visual disturbances. In some cases, PVRL may initially mimic MEWDS, especially in its early stages. However, unlike MEWDS, PVRL occurs more in older or immunocompromised patients, tends to have a progressive course, may be associated with vitritis, and does not resolve spontaneously. Recognizing this overlap is essential, as misdiagnosis can delay appropriate management of PVRL, a potentially life-threatening malignancy [9,10].

## Case presentation

**Fig. 1:**
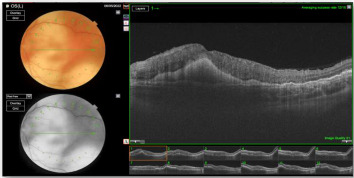
Optical coherence tomography (OCT) of the LE showing multiple subretinal white-yellow lesions, including subfoveal and several nodular subretinal lesions

A 57-year-old male presented with painless, progressive visual loss in the left eye (LE) starting in July 2021. At that time, he was diagnosed at another center with MEWDS and treated with systemic corticosteroids, with subsequent improvement of visual symptoms after a single treatment course. His medical history was significant for COVID-19 infection in September 2021, followed one month later by neurological symptoms including right-hand numbness, right-sided Jacksonian seizures, and balance disturbances. Brain imaging revealed a left frontal tumor, and surgical resection established the diagnosis of primary central nervous system lymphoma (PCNSL) (non-Hodgkin B-cell lymphoma). The patient subsequently underwent systemic and intrathecal methotrexate (MTX) therapy combined with whole-brain radiotherapy. One year later, ophthalmologic reevaluation at our center revealed a best-corrected visual acuity (BCVA) of 20/20 in the right eye (RE) and motion in the left eye (LE). The intraocular pressure (IOP) was normal in both eyes. The LE showed a relative afferent pupillary defect (RAPD), vitreous cells, and multiple subretinal white-yellow lesions, including subfoveal involvement, associated with peripheral pigmentary changes (Fig. 1).

**Fig. 2:**
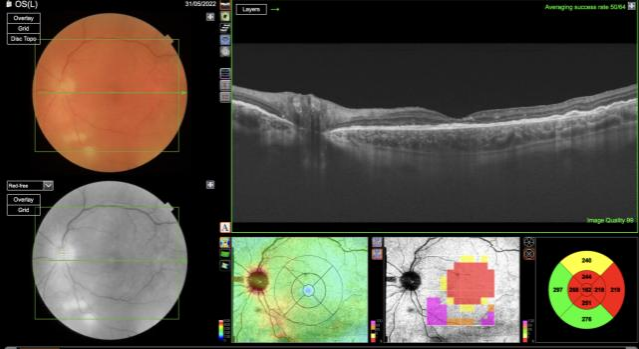
Optical coherence tomography of the LE showing extensive chorioretinal atrophy

Given the patient’s medical history, the findings raised suspicion for intraocular lymphoma. A diagnostic vitrectomy was subsequently performed, confirming PVRL. Intravitreal MTX therapy was initiated. Since the patient was receiving systemic chemotherapy, we considered it prudent to implement a less aggressive therapeutic approach. We started the intensive phase of once-weekly intravitreal injections for 1 month, followed by the consolidation phase of one injection every 2 weeks for 1 month, and then the maintenance phase of once a month for 1 month. No treatment-related complications were observed in this patient. Despite treatment, BCVA in the LE was counting fingers, and extensive chorioretinal atrophy was observed (Fig. 2).

**Fig. 3:**
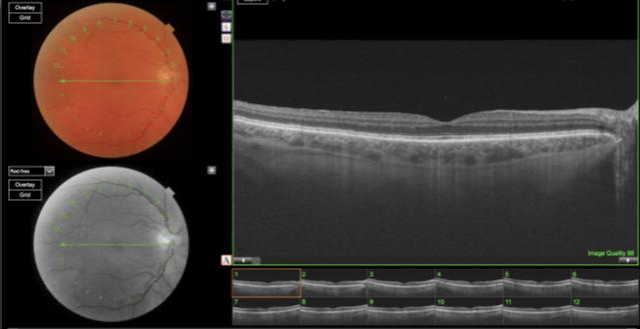
Optical coherence tomography of the RE demonstrating the presence of vitreous cells and mild central pigmentary changes

Although subtle changes in the central outer retinal layers were observed in the RE, BCVA was 20/25. Examination of the posterior pole of the RE revealed the presence of vitreous cells, an infero-temporal retinoschisis, and mild central pigmentary changes (Fig. 3). This situation prompted us to consider the high risk of lymphoma reactivation; therefore, we decided to initiate intravitreal MTX in the RE and to request cerebral imaging along with a hematology consultation. Although the patient underwent an autologous stem cell transplantation in 2023, the clinical course was further complicated by a relapse of PCNSL and a severe Escherichia coli sepsis.

## Discussion

Primary vitreoretinal lymphoma is a rare and aggressive malignancy that often poses a significant diagnostic challenge because it can mimic a wide range of inflammatory and infectious ocular conditions [11]. Presenting PVRL as a WDS in our report underscored its reputation as a “great masquerader”. White dot syndromes are typically considered benign, self-limiting inflammatory disorders; therefore, an initial misdiagnosis may lead to delays in appropriate investigation and treatment [12]. The overlap in clinical features - such as subretinal lesions, vitreous inflammation, and visual disturbances - can obscure the underlying neoplastic process, particularly in the early stages of disease [13]. A key consideration highlighted by this case is the importance of maintaining a high index of suspicion when clinical findings are atypical, recurrent, or unresponsive to conventional therapy. Multimodal imaging, including fundus photography, fluorescein angiography, and OCT, can provide valuable clues, such as subretinal infiltrates and characteristic patterns that are not entirely consistent with inflammatory etiologies [14]. However, definitive diagnosis typically requires cytological analysis of vitreous or retinal tissue, often supplemented by immunohistochemistry and molecular studies [15].

Multiple evanescent white dot syndrome is generally not considered in the differential diagnosis of masquerade syndromes because it is typically an acute, benign, and self-resolving condition [16]. Nevertheless, PVRL can, in rare instances, imitate its clinical appearance. Reported cases describe PVRL presenting with gray-white outer retinal lesions that appear hyperautofluorescent on fundus autofluorescence (FAF) and show ellipsoid zone disruption on OCT, closely resembling MEWDS. In other cases, OCT findings may be more indicative of lymphoma, demonstrating retinal pigment epithelium (RPE) elevation due to sub-RPE hyperreflective deposits, with possible extension into the outer nuclear layer. Features that may help distinguish lymphoma from MEWDS include older patient age, the presence of vitritis, absence of spontaneous resolution, and the characteristic mottled pattern of hypo- and hyperfluorescence (“leopard spots”) observed on fluorescein angiography and FAF [16]. Ultimately, a definitive diagnosis relies on cytologic confirmation obtained through vitreous biopsy and/or lumbar puncture [17]. In our case, the initial presentation was not fully documented with OCT, and therefore, no baseline reference was available.

The differential diagnosis in such cases is broad and includes infectious retinitis (viral, toxoplasmic, or syphilitic), non-infectious uveitic entities such as sarcoidosis, and other masquerade syndromes, including intraocular metastases [18]. Distinguishing PVRL from these conditions is critical, as management strategies differ substantially [19]. Given the strong association between PVRL and PCNSL, delayed diagnosis not only prolongs visual morbidity but also has systemic implications. Therefore, early recognition and timely referral for systemic evaluation, including neuroimaging and cerebrospinal fluid analysis, are essential components of patient care [20].

The standard treatment protocol for PVRL consists of intravitreal MTX injections administered twice weekly for 4 weeks, then once weekly for 8 weeks, and subsequently once monthly for 9 months, for a total of 25 injections. Zhou et al. [21] demonstrated that complete clinical remission can be achieved with fewer injections, thereby improving treatment compliance and reducing the incidence of complications. Therefore, in our case, we adopted the following protocol: an intensive phase consisting of once-weekly intravitreal injections for 1 month, followed by a consolidation phase of one injection every 2 weeks for 1 month, and then a maintenance phase of once-monthly injections for 1 month, for a total of 7 injections. The patient did not experience any treatment-related complications. Furthermore, the patient’s life expectancy was not adversely affected during the follow-up periods.

The diagnosis of PVRL is frequently delayed, often because patients present to an ophthalmologist late. Many individuals initially report persistent floaters that impair visual quality despite relatively preserved visual acuity, potentially contributing to under-recognition. Reported mortality rates in the literature vary widely, reflecting differences in patient populations, treatment approaches, and the small size of most case series. Across studies, mortality has been reported to range from 9% to 81% over follow-up periods of 12 to 35 months [15,19,22,23]. In cases of PCNSL, median survival for patients treated with radiotherapy, with or without chemotherapy, has been reported between 10 and 16 months, but may exceed 30 months with methotrexate-based regimens or agents such as ifosfamide or trofosfamide [2,4]. In a large multicenter study, Grimm et al. [2,5] reported a median progression-free survival of 18 months and an overall survival of 31 months among patients with PCNSL and intraocular involvement treated with various therapeutic strategies, concluding that while ocular treatment improved local disease control, it did not significantly impact overall survival. Conversely, other studies have suggested a survival benefit when PVRL is diagnosed before CNS involvement, with reported survival of 60 months compared to 35 months, although patients diagnosed earlier tended to be younger [2,6].

This case reinforces the need for vigilance in patients with presumed WDS who follow an unusual clinical course. Ophthalmologists should consider PVRL in the differential diagnosis, particularly in older patients or in those with persistent or progressive disease despite standard therapy. Early diagnostic intervention, including vitreous biopsy when indicated, can facilitate prompt initiation of appropriate oncologic treatment and improve overall prognosis. Ultimately, greater awareness of atypical presentations of PVRL may help reduce diagnostic delays and optimize both ocular and systemic outcomes.

## Conclusion

Primary vitreoretinal lymphoma should be considered in the differential diagnosis of white dot-like retinal lesions, especially in patients with a history of CNS lymphoma. Recognition of this masquerade presentation is essential to avoid diagnostic delay and to ensure prompt multidisciplinary management. Early suspicion and appropriate diagnostic workup, including ocular imaging and cytologic analysis, are critical for confirming the diagnosis. Timely intervention may significantly influence both visual prognosis and overall patient survival.
